# Applications of Fourier Transform Ion Cyclotron Resonance (FT-ICR) and Orbitrap Based High Resolution Mass Spectrometry in Metabolomics and Lipidomics

**DOI:** 10.3390/ijms17060816

**Published:** 2016-05-25

**Authors:** Manoj Ghaste, Robert Mistrik, Vladimir Shulaev

**Affiliations:** 1 Department of Biological Sciences, College of Arts and Sciences, University of North Texas, Denton, TX 76203, USA; manoj.ghaste@unt.edu; 2 HighChem, Bratislava 81104, Slovakia; robert.mistrik@highchem.com

**Keywords:** high resolution mass spectrometry, metabolomics, lipidomics, FTMS, FT-ICR-MS, Orbitrap-MS, metabolomics data analysis

## Abstract

Metabolomics, along with other “omics” approaches, is rapidly becoming one of the major approaches aimed at understanding the organization and dynamics of metabolic networks. Mass spectrometry is often a technique of choice for metabolomics studies due to its high sensitivity, reproducibility and wide dynamic range. High resolution mass spectrometry (HRMS) is a widely practiced technique in analytical and bioanalytical sciences. It offers exceptionally high resolution and the highest degree of structural confirmation. Many metabolomics studies have been conducted using HRMS over the past decade. In this review, we will explore the latest developments in Fourier transform mass spectrometry (FTMS) and Orbitrap based metabolomics technology, its advantages and drawbacks for using in metabolomics and lipidomics studies, and development of novel approaches for processing HRMS data.

## 1. Introduction

Metabolomics is a global approach aimed at measuring cell metabolomes, which are “context dependent, varying according to the physiology, developmental or pathological state of the cell, tissue, organ or organism” [1]. Metabolomics experiments generally target a large number of chemically diverse small molecular weight compounds including primary metabolites, such as organic acids, amino acids, sugars, sugar alcohols, sugar phosphates, amines, fatty acids, polar lipids, hormones and vitamins, as well as specialized metabolites, like phenolics, flavonoids, monoterpenes, sesquiterpenes, polyketides, alkaloids, and others. Lipidomics aims at measuring a full complement of lipid molecular species in cells, tissues, or organisms [2]. There is an overlap in metabolites usually covered by metabolomics and lipidomics (*i.e.*, many polar lipids, fatty acids, eicosanoids and fat soluble vitamins), and some scientists consider lipids as a subset of metabolome and lipidomics as part of metabolomics. In recent years, metabolomics and lipidomics have become the major analytical approaches in many areas of biology ranging from studying gene functions to systems biology research complementing genomics, transcriptomics and proteomics approaches aimed at understanding global state of the cell.

Mass spectrometry (MS) is often the technique of choice to generate high-throughput metabolomics and lipidomics data due to high sensitivity, relatively short analysis time, wide dynamic range, high reproducibility and, most importantly, its ability to analyze samples with extreme molecular complexity [3,4,5,6]. Over the years, MS has proven itself as powerful technology for the detection and annotation of diverse metabolite classes and has become an important tool for metabolomics analysis in numerous organisms [7]. Therefore, various conventional MS based multiclass analyses are now being replaced by metabolomics approaches that offer excellent combinations of analytical and bioinformatics tools and can provide comprehensive information on a large number of metabolites in any particular system.

Fourier transform mass spectrometers (FTMS) or Fourier transform ion cyclotron resonance mass spectrometers (FT-ICR-MS) are the most advanced mass analyzers in terms of high accuracy and resolving power with sub-parts-per-million mass accuracy [8]. The FT based mass analyzers principally use cyclotron frequency in the fixed magnetic field for the determination of the ions mass to charge ratio (*m*/*z*) [9,10] and provide the resolution and mass accuracy that are required to reliably assign molecular formulas to detected ions [11]. The accurate mass measurement by FTMS has been widely demonstrated for the characterization of unknown metabolites by the unambiguous assignment of elemental formulas [3,10,12,13,14,15]. These characteristics of FTMS are ideal for the types of complex mixtures encountered in high throughput metabolomics applications [13].

The disadvantage of FT-ICR-MS instruments is their relatively slow acquisition rates. At a scan rate of 1 Hz with mass resolution of 100,000 at *m*/*z* 4000, the number of points over the chromatographic peak, especially if additional MS/MS scans are required, is low when FTMS is combined with modern fast chromatography systems. This limits the application of FT-ICR-MS in liquid chromatography mass spectrometry (LC-MS) and capillary electrophoresis mass spectrometry (CE-MS) based metabolomics. Introduction of the Orbitrap mass analyzer [16] and coupling Orbitrap with liquid [17] and later gas [18,19] chromatography resulted in a growing number of studies employing high resolution mass spectrometry (HRMS) in metabolomics and lipidomics.

Metabolomics [13,20,21] and lipidomics [22] applications of FT-ICR-MS and Orbitrap-MS have been previously reviewed. In this short review, we provide a critical overview of the latest developments in the HRMS based metabolomics approach and its potential for metabolomics, lipidomics and high-density pharmaceutical and environmental analysis. We will mostly focus on applications of FT-ICR-MS and Orbitrap instruments.

## 2. Advantages of Fourier Transform Ion Cyclotron Resonance (FT-ICR) and Orbitrap Based High Resolution Mass Spectrometry (HRMS) for Metabolomics and Lipidomics

High Resolution Mass Spectrometers can routinely achieve mass accuracy below 5 ppm and a mass resolution above 10,000 (which is ratio of measured mass (m) to theoretical mass (Δm) m/Δm, at the full width of the peak at half of its maximum height (FWHM)) (reviewed by [20]). Mass analyzers that can perform HRMS are FT-ICR, Orbitrap and time of flight (TOF) analyzers. TOF based instruments can achieve mass resolution up to 60,000 (at *m*/*z* 200), while Orbitrap based instruments can achieve much higher mass resolution—up to 240,000, and over 1,000,000 for FT-ICR (at *m*/*z* 400) [20]. HRMS provide several advantages in metabolomics and lipidomics studies, including high resolving power, increased mass accuracy and increased limits of detection [13]. High resolution and mass accuracy also allows for adduct identification with high precision [13]. Application of HRMS based DNA adductomics describing screening of known and unknown adducts of the DNA by using Orbitrap based multiple-stage mass spectrometry (MS^n^, n = tandem stage) experiments was recently reported [23,24]. Due to these advantages, HRMS techniques are being increasingly used in metabolomics. Below, we summarize major advantages of FT-ICR and Orbitrap based instruments for metabolomics and lipidomics studies by HRMS:

(a) High mass resolution with the ability to achieve measurements with ppm and sub-ppm errors allows a complex metabolic extract to be analyzed with minimal chance of interference from overlap of other species in the mass spectrum [13]. The ability to discriminate metabolites at the 1–3 ppm level not only dramatically improves characterization of complex mixtures but also minimizes ambiguity of molecular formula assignments.
(b) Extremely high mass accuracy and sufficiently high acquisition rates makes FT-ICR and Orbitrap based instruments very popular in direct infusion mass spectrometry (DIMS), especially for metabolic and lipidomics fingerprinting studies. The ability of direct sample infusion would be clearly advantageous over existing time-consuming metabolite analyses or screening methods. With only a few minutes required for data acquisition with very high information content [12], DIMS can decrease the demand for extensive chromatographic separation and dramatically increase sample throughput in large scale screening experiments [12,25,26]. The high-throughput approach permits a sample to be processed within a few minutes and the short analysis time increases inter-sample reproducibility and improves the accuracy of subsequent cluster analysis [27].
(c) Flexibility in choosing the ion source or fragmentation technique. Variety of ion sources, including electrospray ionization (ESI), atmospheric pressure chemical ionization (APCI), vacuum or atmospheric, matrix assisted laser desorption ionization (MALDI), desorption ionization (DESI), and direct analysis in real time (DART), are currently available and have been used for metabolomics and lipidomics applications [5,6,13,20,28,29]. Different fragmentation techniques like collision induced dissociation (CID), higher-energy collisional dissociation (HCD), electron induced dissociation (EID), infrared multiphoton dissociation (IRMPD), electron-transfer dissociation (ETD) and electron-transfer and higher-energy collision dissociation (EThcD) are available at any stage of MS^n^ with detection in either the Orbitrap or linear ion trap detector [20].
(d) Ability to perform stepwise fragmentation in multiple stage mass spectrometry (MS^n^) experiments is useful for more confident metabolite identification or in-depth metabolite characterization of unknown compounds [7].

## 3. HRMS Applications in Metabolomics and Lipidomics

Numerous studies have been published employing HRMS based techniques in metabolomics [30,31,32,33,34,35,36,37,38,39,40,41], lipidomics [2,22,42,43,44,45,46,47,48,49,50,51,52,53,54,55,56,57,58,59,60,61,62,63,64,65,66,67], glycomics [68,69,70], food chemistry [71,72,73], natural products discovery [74,75], environmental [76,77] and pharmaceutical [78,79] studies.

HRMS based metabolomics and lipidomics can be performed either by a shotgun approach based on DIMS where samples are directly infused into a mass spectrometer or using chromatographic or electrophoretic separation prior to MS detection.

### 3.1. Shotgun Based Approaches

Shotgun approaches based on DIMS are being widely used due to their simplicity, limited sample prep and high throughput. Additionally, the data from direct infusion experiments can be directly used in multivariate statistical analysis without complicated data pre-processing steps. DIMS was successfully applied to metabolomics and lipidomics studies using both FT-ICR and Orbitrap analyzers.

DIMS provides several advantages to large scale metabolomics studies where analysis speed and sample throughout is most important. High mass accuracy and resolution of FT-ICR and Orbitrap instruments can significantly increase the number of molecular species detected in fingerprinting experiments. Even though many metabolites can be observed in DIMS, the majority of the ions remain unidentified. Therefore, this approach is often used in metabolic or lipidomic fingerprinting experiments [80].

Aharoni and co-authors [12] used a direct infusion FTMS approach for high throughput metabolic screening of differentially expressed metabolites in a mutant strawberry population with a relative quantitation and putative identification. Since different isomers sometimes show identical empirical formulas, the data obtained by FTMS experiment was further correlated with data obtained from gene expression studies using DNA microarrays [12]. Authors also suggested the use of preferably similar matrices to avoid any ion suppression and elimination of the adduct formation. In another study, Witting and co-authors [31] used direct-infusion ion-cyclotron-resonance Fourier-transform mass spectrometry (DI-ICR-FT-MS) in non-targeted metabolomics to obtain high-resolution snapshots of the metabolic state of a *Caenorhabditis elegans* interacting with pathogens. They identified marked decrease in amino-acid metabolism with infection by *Pseudomonas aeruginosa* and a marked increase in sugar metabolism with infection by *Salmonella enterica*.

The shotgun approach, in the case of lipidomics, is proven to be an effective method to get quick snapshots of molecular composition of complex lipidomes, and this approach has been demonstrated successfully in combination with HRMS [2,22,42,49,51,58,59,62,81,82,83,84,85,86]. The Orbitrap mass spectrometers are especially useful in shotgun lipidomics because of their rapid acquisition of MS/MS spectra, higher mass resolution and optional MS^n^ fragmentation [2,42,44], and, most importantly, the rapid polarity switching with sub-ppm mass accuracy, which ultimately simplifies and accelerates the shotgun lipidomics analysis and improves lipidome coverage [42].

Matrix assisted laser desorption ionization (MALDI) coupled to FTMS is another approach that can be used for shotgun analysis. This technique, for example, was used by Wang and co-authors to study urinary metabolites, mainly focused on prediction of acute cellular renal allograft rejection [87] and acute tubular injury [88] through urinary metabolomics. Both studies suggest that the use of MALDI resulted in production of singly charged species and higher sensitivity and specificity [87,88].

Despite many advantages, DIMS and other shotgun approaches suffer significant drawbacks mostly related to their limited ability to resolve isobaric species or co-suppression effect where useful signals from many metabolites are lost at the mass spectrometer interface. To minimize co-suppression effect, two-step fingerprinting/validating strategy [89] or fractionated fingerprinting approach [90,91] can be used for metabolic fingerprinting.

### 3.2. Hyphenated Techniques

To overcome issues related to sample complexity, co-suppression, and improve resolution of isobaric species, FTMS is often used in combination with front-end chromatography or electrophoresis separation techniques like gas chromatography (GC), liquid chromatography (LC), ion chromatography (IC) or capillary electrophoresis (CE) (reviewed by [21,92]).

LC-MS analysis has been extensively used in metabolomics and lipidomics studies over the last decade [93,94,95,96,97,98,99,100,101,102,103,104,105,106,107,108,109,110,111,112,113,114,115,116,117,118]. It offers high sensitivity, high resolution and covers wide polarity and molecular weight range of analytes. Over the years, a large number of LC-MS based techniques have been developed to study many metabolite classes.

Recent advances in LC separation methodology, including development of ultra-performance liquid chromatography (UPLC), using capillary monolithic columns, and application of fused core particles, significantly improved chromatographic resolution and resulted in increased analysis speed and metabolite coverage, which is critical in large scale metabolomics experiments.

Application of solid or fused-core particles can provide faster chromatographic separation and increased sample throughput. Hu and co-authors [93] demonstrated the development and validation of the LC-FT-ICR-MS method for profiling of lipids in human and mouse plasma using a fused-core column. They used a C8 column with 2.7 µm fused-core silica particles and a 0.5 µm thick porous shell, which allows higher flow rates and faster separations that are subsequently detected by FTMS [93]. In a recent study, Granafei and co-authors [119] demonstrated the use of fused-core ultrapure silica particles (2.7 µm) narrow bore column in combination with LC-ESI-FTMS for the identification of isobaric lyso-phosphatidylcholines (LPC) in lipid extract of gilthead sea bream, which led to significant improvement in chromatographic resolution of phospholipids and, in combination with Orbitrap MS, it was useful to resolve the remarkable complexity of LPC [119]. Solid or fused core particles are now available in many different phases and particle sizes (from 1.6 to 5 µm) and are provided by multiple commercial vendors. Damen and co-authors described a novel approach for the separation of different lipid molecular species and lipid isomers in human plasma using a stationary phase incorporating charged surface hybrid (CSH) technology using reversed-phase UPLC combined with ion-mobility and HRMS [120].

To increase metabolite coverage, it is plausible to use multiple chromatographic separations utilizing different column chemistry and combine data from these separations. Soltow and co-authors, for example, used a dual chromatography-Fourier-transform mass spectrometry (DC-FTMS) approach to increase the number of detected metabolites in their study of the exposome [26]. Authors performed sequential LC-FTMS analyses using reverse phase (C18) chromatography and anion exchange (AE) chromatography. This approach increased *m*/*z* feature detection by 23%–36%, yielding a total number of features up to 7000 for individual samples when compared to analysis with the AE column alone. From all detected features, approximately 50% of the *m*/*z* was matched to known chemicals in metabolomic databases, and 23% of the *m*/*z* were common to analyses on both columns.

Significant technical advances for the HRMS-based metabolomics in the past few years was the introduction of the GC-enabled quadrupole linear ion trap (QLT)-Orbitrap hybrid mass spectrometer capable of high resolution (up to 100,000 at *m*/*z* 400) and sub-parts-per-million mass accuracy GC-MS [121]. The performance of the new instrument was demonstrated by its application to the determination of polychlorinated dibenzo-p-dioxins and dibenzofurans in the environmental samples and profiling of primary metabolites in *Arabidopsis thaliana* extracts [121]. Later, Peterson and co-authors [18,19] reported the development of GC/Quadrupole Orbitrap mass spectrometer which combines high mass accuracy, high resolution, and high sensitivity analyte detection that makes it a promising instrument for both untargeted and targeted metabolomics studies. The authors also developed an “intelligent” data-dependent algorithm, termed molecular ion directed acquisition (MIDA). This algorithm maximizes the information content generated from unsupervised tandem MS and selected ion monitoring (SIM) by directing the MS to target the ions of greatest information content [18]. New instruments and software were successfully used for non-targeted metabolomics. Combination of ^13^C- and ^15^N-metabolic labeling, multiple derivatization and ionization types, and heuristic filtering of candidate elemental compositions allowed to achieve MS/MS spectra of nearly all intact ion species for structural elucidation, knowledge of carbon and nitrogen atom content for every ion in MS and MS/MS spectra, relative quantification between alternatively labeled samples, and unambiguous annotation of elemental composition [18,19]. This proved it to be a very promising technology in discovery metabolomics to study volatile compounds or compounds that can be volatilized by chemical derivatization.

### 3.3. Mass Spectrometry Imaging

One of the drawbacks of the DIMS and most hyphenated techniques is their inability to provide spatial information on localization of various metabolites and lipids in organs and tissues. The mass spectrometry imaging (MSI) approach can provide this spatial information. Multiple MSI approaches based on different ionization techniques have been developed and are currently being widely used. Among them, MALDI combined with HRMS is the most often used in imaging applications. It has been successfully used for imaging a variety of human [122,123,124,125,126], animal [127,128,129,130,131] and plant [132,133] tissues. For example, spatial mapping of lipids at cellular resolution in cotton embryos using MALDI Hybrid Ion Trap-Orbitrap (MALDI LTQ Orbitrap XL) mass spectrometer showed differential distribution of lipid species such as triglycerols and phosphatidylcholines [133,134]. Many other lipidomics applications of MALDI imaging were subsequently reviewed by [133,134,135]. Most MSI applications are currently focused on imaging lipids [136,137,138,139,140,141,142,143], although imaging of primary and specialized metabolites and xenobiotics has been reported [122,128,132,144,145,146].

Ambient ionization methods, such as atmospheric pressure MALDI (AP-MALDI) [147,148,149,150,151], desorption electrospray ionization (DESI) [152,153] and matrix-assisted laser desorption electrospray ionization (MALDESI) [146,154,155,156], have also been employed for MSI. Ambient ionization approaches can provide certain advantages over the vacuum MALDI source for MSI. For example, AP-MALDI can provide high spatial resolution (below 10 µm) with high mass resolution and high mass accuracy obtained with Orbitrap-based instrumentation [147,148,149,150,151].

### 3.4. Other Applications of HRMS

Analytical advantages of HRMS make it broadly applicable to other fields beyond metabolomics and lipidomics. Numerous studies have been published employing HRMS based techniques in many research areas, such as glycomics [68,69,70], food science [71,72,73], forensics [157], toxicology [157,158], natural products discovery [74,75], agriculture [37,96,159,160,161,162,163,164,165,166,167], environmental [76,77] and pharmaceutical [78,79] studies. In recent years, food authenticity and safety have become a global concern prompting the development of novel analytical techniques to address food safety issues. The role of HRMS in many studies has proven it to be crucial for understanding process contamination, food adulteration and food contaminants, such as pesticides and mycotoxins [71,72,159,160,161,162]. In a case study of doping control, Kiss and colleagues used ultra HRMS based non-targeted metabolomics to study salbutamol and budesonide abuse through analysis of human urinary metabolites [168]. In another doping control study, markers of testosterone misuse were analyzed by untargeted metabolomics approach and HRMS [79]. Applications of HRMS in agriculture range from determining mycotoxins in agricultural products [163,164,165] and profiling human health related metabolites in crop plants [169,170,171] to studying the effect of different diets on animal metabolism [37]. In recent study, Sun and co-authors studied the effect of high fat, high cholesterol diet on changes in metabolite patterns in pigs [37]. They analyzed plasma, fecal and urine samples from pigs fed high fat or basal regular diets using Ultra High Performance Liquid Chromatography (UHPLC)-HRMS and chemometric analysis and found a set of metabolites most affected by the diet [37,172]. Although the application of metabolomics in environmental studies for the analysis of environmental pollutants has been reported [164,167,173,174,175,176,177,178,179], the use of HRMS based MS techniques for environmental research is still limited [76,77]. Applications of mass spectrometry in the pharmaceutical metabolomics can be further expanded by using HRMS (reviewed by Drexler and colleagues [78]).

## 4. Data Analysis and Databases

Metabolomics experiments based on non-targeted HRMS analysis generate large amounts of data and require extensive raw data pre-processing and application of specialized mathematical, statistical and bioinformatics tools [92,180,181,182,183]. Pre-processing can be done by using in-house or specialized tools [184,185]. Multivariate statistics tools commonly used to analyze metabolomics data include pattern recognition, identification of outliers, reduction of data dimensionality, and compression of large datasets [92,182,186].

As FTMS provides the resolution and mass accuracy that are required to reliably assign molecular formulas to detected ions, it is imperative to use this information to metabolite identifications. It is generally accepted that accurate mass alone is not sufficient to positively identify an unknown structure, and the chemical structure database returns multiple hits (sometime several hundred or more) at a defined mass tolerance window. Even at the highest mass resolution, FTMS cannot provide exact identification because many isobaric species and structural isomers have identical empirical formulas. In such cases, additional information, including chromatographic retention time, isotope pattern matching, collisional cross section (CCS), and use of multiple stage mass spectrometry (MS^n^) is needed for correct compound annotation [187,188,189]. MS data can also be correlated with nuclear magnetic resonance (NMR) data.

Numerous chemical reference databases and mass spectral libraries are currently publicly available. There is also a significant increase in accurate mass enabled mass spectral databases in recent years.

Reference chemical and biochemical databases are either focused on collecting reference information on chemical compounds independently of their sources or provide information on endogenous and exogenous metabolites linked to a particular biological system or matrix. Some databases are limited to a particular metabolite classes. Among the largest chemical databases are PubChem [190,191,192] and ChemSpider [193,194,195]. Mass spectral libraries and databases containing fragmentation data are invaluable resources for compound identification. Until recently, many MS/MS databases contained only nominal mass spectral data. The increased application of HRMS in metabolomics leads to the development of accurate mass enabled spectral search programs and databases that contain information on accurate mass of just the precursor or both the precursor and fragment ions. The popular NIST (National Institute of Standards and Technology) MS Search program version 2.0 released in 2011 (Standard Reference Data, NIST, Gaithersburg, MD, USA) allows for exact mass search of parent and fragment ion. Many open-access spectral databases also contain accurate mass information and high resolution mass spectra. For example, the Scripps Center for Metabolomics released the online database called METLIN [196] which is a repository of metabolite information and tandem mass spectrometry data designed to facilitate metabolite identification in metabolomics [33,197,198,199]. The database provides comprehensive MS/MS metabolite data and each metabolite is linked to outside resources like Kyoto Encyclopedia of Genes and Genomes (KEGG) for further reference. The webserver MassTRIX [200,201,202] provides assignment of the bulk chemical formulae considering biological and genomic context of the samples. The mass difference network based approach for formula calculation [203] or on data combination from lower resolution LC-MS with FT-ICR-MS [204] can also be used.

Other metabolomics databases used in many metabolomics studies include KEGG [205,206], Madison Metabolomics Consortium Database (MMCD) [207], Human Metabolome Database and drug bank [208,209], and LIPID Metabolites and Pathway Strategy (LIPIDMAPS) [210,211]. Tools for putative metabolite identification using multiple online databases, e.g., MetaboSearch, can simplify concurrent searches of multiple metabolite databases [212].

**Figure 1:**
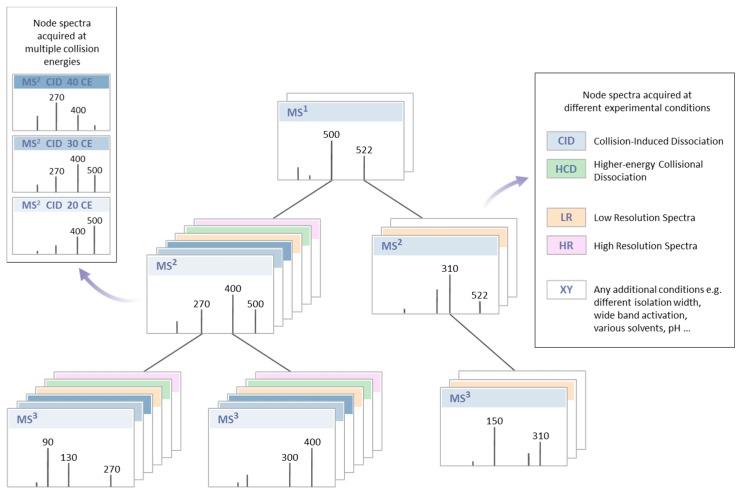
In tandem mass spectrometry, a spectral tree is a data structure encapsulating the hierarchically organized product ion mass spectra of a single chemical compound where each level represents an MS^n^ stage. Edges refer to precursor *m*/*z* values, and nodes refer to spectra generated from the particular precursor ions.

**Figure 2:**
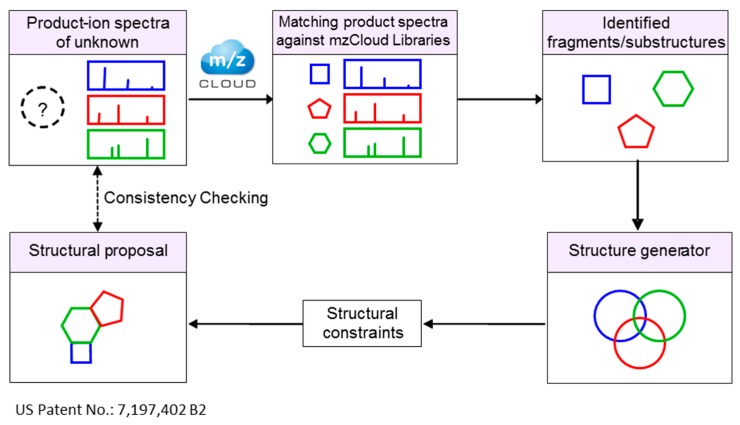
Strategy for structure elucidation for unknown metabolites using mzCloud library of mass spectral fragments/substructures.

Specialized algorithms for profiling individual metabolite classes have also been developed [213,214]. For example, the program “LipidSearch” developed by Taguchi and co-authors [213], utilizes specific detection approach by neutral loss survey-dependent MS^3^, for the identification of molecular species of phosphatidylcholine, sphingomyelin and phosphatidylserine. LipidSearch program combined with HRMS was successfully used for lipid annotation in multiple studies [213,215,216,217,218,219]. Several other programs such as LipidQA [220], LIMSA [221], FAATc [222], lipID [223], LipidView [50], LipidInspector [46] (Herzog *et al.*) are specialized in identification of lipids from the shotgun experiments. Extensive review of bioinformatics tools and software can be found in the literature [44,224]. A novel approach to represent and calculate the similarity between high-resolution mass spectral trees (Figure 1) has been proposed for the construction of the MS^n^ libraries for the annotation and structural elucidation of the unknown metabolites. Structures of the unknown metabolites can be predicted in a high throughput approach utilizing fragmentation trees and precursor ion fingerprinting (PIF) technique [7,225,226]. This approach utilizes structural information from high resolution fragmentation spectra and predicts the identity of the unknown metabolite by determining its possible substructures (Figure 1). High resolution MS^n^ spectra of unknown metabolites are first collected using the spectral ion tree approach. The product ion MS^n^ spectra of various precursor ions are then searched against the MS^n^ spectral library. Although the unknown metabolite is not present in the spectral library, the spectra of molecules that contain similar substructures will provide unambiguous substructure identifications. Identified substructures/fragments are used to generate a structural proposal for unknown metabolite (Figure 2).

Until recently, wider use of this approach was limited by the lack of publically available curated spectral tree databases. The newly developed mzCloud library [227] provides a significant step forward in this direction. mzCloud is a novel type of mass spectral database that can help to predict structures of unknown metabolites and identify compounds even when they are not present in the mass spectral library using PIF technique [226]. This is of immense value when traditional library search yields no results. PIF relies on well defined and chemically plausible structures of fragment ions, which are either used to reassemble the parent compound or, at the very least, point towards its structural characteristics. The library contains substructurally characterized precursor ions of MS^n^ spectra calculated using heuristic and quantum chemical methods. The quantum chemical annotation pipeline for precursor ion prediction contains over 500,000 unique 3D structures with calculated thermochemical properties in semi-empirical and Discrete Fourier Transform (DFT) levels of theory. Each spectral peak present in the library is annotated by one or more alternative molecular formulas that can be displayed in the spectra. mzCloud employs a freely searchable collection of manually curated, high resolution/accurate mass spectra based on the cloud technology. mzCloud library has an advanced database viewer which displays: spectral trees, MS^n^ spectra, structures, fragments, fragmentation patterns, collision energies, resolution, accuracy, isolation width, names, break-down curves and other relevant information. To date, the mzCloud database features over 1,00,000 processed spectral records covering a wide range of collision energies up to MS^8^ in 4300 endogenous metabolites, plant secondary metabolites, food additives, pharmaceuticals, environmental contaminants and other compounds relevant for metabolomics.

## 5. Conclusions: Technological Developments and Future Perspectives

FTMS and Orbitrap based MS technology have proven to be useful for untargeted or targeted screening and a broad range of qualitative and quantitative applications in diverse fields like metabolomics, lipidomics, drug discovery, proteomics, environmental and food safety, clinical research, forensic toxicology and agricultural science. Today, we have some of the best and highly advanced FTMS and Orbitrap systems available on the market. Modern FTMS systems offer ultra-high mass resolution of 10,000,000, while current Orbitrap systems offer less than 1 ppm mass accuracy, up to 450,000 FWHM, more than four orders of magnitude intrascan dynamic range, along with femtogram-level sensitivity, a fast scanning rate at 15 Hz and spectral multiplexing suited to UHPLC applications and mass range to 6000 Da. Improvement in instrumentation was accompanied by development of new data processing algorithms, software and databases, many of which are available in the public domain.

In the near future, we should see more improvements in instrumentation and processing software, specifically in increasing data acquisition rate, improving isotope ratio accuracy, exploring more hyphenated techniques and multi-dimensional chromatography, and creating integrated and flexible data processing solutions from raw data to biological interpretation. It is also necessary to increase community efforts in standardizing metabolomics and lipidomics data and metadata standards, and expanding bioinformatics tools and metabolomics data repositories available to the community.

## Abbreviations

The following abbreviations are used in this manuscript:

AE	Anion exchange
APCI	Atmospheric pressure chemical ionization
AP-MALDI	Atmospheric pressure matrix-assisted laser desorption ionization
ATI	Acute tubular injury
CCS	Collisional cross section
CE-MS	Capillary electrophoresis mass spectrometry
CID	Collision induced dissociation
CSH	Charged surface hybrid
DART	Direct analysis in real time
DC-FTMS	Dual chromatography-Fourier transform mass spectrometry
DESI	Desorption electrospray ionization
DFT	Discrete Fourier transform
DIMS	Direct infusion mass spectrometry
DNA	Deoxyribonucleic acid
EID	Electron induced dissociation
ESI	Electro spray ionization
ETD	Electron-transfer dissociation
EThcD	Electron-transfer and higher-energy collision dissociation
FT-ICR-MS	Fourier transform ion cyclotron resonance mass spectrometry
FTMS	Fourier transform mass spectrometry
FWHM	Full width at half maximum
GC-MS	Gas chromatography coupled to mass spectrometry
HCD	Higher-energy collisional dissociation
HRMS	High resolution mass spectrometry
IC	Ion chromatography
IRMPD	Infrared multiphoton dissociation
KEGG	Kyoto encyclopedia of genes and genomes
LC-FT-ICR-MS	Liquid chromatography-Fourier transform ion cyclotron resonance mass spectrometry
LC-FTMS	Liquid chromatography-Fourier transform mass spectrometry
LC-MS	Liquid chromatography coupled to mass spectrometry
LPC	Lyso-phosphatidylcholines
*m*/*z*	Mass to charge ratio
MALDESI	Matrix-assisted laser desorption electrospray ionization
MALDI	Matrix assisted laser desorption ionization
MIDA	Molecular ion directed acquisition
MMCD	Madison metabolomics consortium database
MS/MS	Tandem mass spectrometry
MS	Mass spectrometry
MSI	Mass spectrometry imaging
MS^n^	Multiple-stage mass spectrometry
NMR	Nuclear magnetic resonance
PCDD	Polychlorinated dibenzo-p-dioxins
PCDF	Polychlorinated dibenzofurans
PIF	Precursor ion fingerprinting
SIM	Selected ion monitoring
UHPLC	Ultra high performance liquid chromatography
UPLC	Ultra performance liquid chromatography
